# Carpal tunnel surgery: predictors of clinical outcomes and patients’ satisfaction

**DOI:** 10.1186/s12891-020-3082-2

**Published:** 2020-01-28

**Authors:** Ehsan Alimohammadi, Seyed Reza Bagheri, Homa Hadidi, Parisa Rizevandi, Alireza Abdi

**Affiliations:** 1 Department of Neurosurgery, Kermanshah University of Medical Sciences, Imam Reza hospital, Kermanshah, Iran; 2 Department of Neurosurgery, University of Medical Sciences, Imam Reza hospital, Kermanshah, Iran; 3 Clinical Research Development Center, Taleghani and Imam Ali Hospital, Kermanshah University of Medical Sciences, Kermanshah, Iran; 4 operating room department, Kermanshah University of Medical Sciences, Imam Reza hospital, Kermanshah, Iran; 5 nursing and midwifery school, Kermanshah University of Medical Sciences, Imam Reza hospital, Kermanshah, Iran

**Keywords:** Carpal tunnel syndrome, Clinical outcomes, Patients’ satisfaction, Boston carpal tunnel questionnaire, Symptom severity scale, Functional status scale

## Abstract

**Background:**

Carpal tunnel syndrome (CTS) is the most common peripheral neuropathy. Moreover, carpal tunnel release (CTR) surgery generally has excellent results. The present study aimed to investigate the predictors of clinical outcomes and satisfaction in patients with CTR.

**Methods:**

In this observational prospective cohort study, 152 patients with open carpal tunnel release surgery were investigated. Complete clinical examinations were performed and recorded before the surgery, two weeks after the surgery and 6 months after the surgery. The Boston Carpal Tunnel Questionnaire (BCTQ) were assessed on admission and at last follow-up visits to evaluate clinical outcomes. Patients’ satisfaction was determined by a 10-point verbal descriptor nominal scale (1 = very poor, 5 = fair and 10 = excellent) and recorded during the last follow -up visits.

**Results:**

Among 152 patients who were investigated, there were 118 (77.6%) females and 34 (22.36%) males. Overall, surgery improved the outcomes based on Symptom Severity Scale (SSS) and Functional Status Scale (FSS) (*P* < 0.05). Most of the considered variables did not show significant effects on clinical outcomes and patients’ satisfaction. However, duration of symptoms and electrophysiological severity were the predictors of the change score in SSS(*P* < 0.05). As well as, age was the only predictor of the change score in FSS (*P* < 0.05). Finally, according to the linear regression model, the pre-operative grip strength and age were the independent predictors of post-operative satisfaction (*P* < 0.05).

**Conclusions:**

Results of the present study revealed that there was a significant improvement in clinical outcomes after CTS surgery. Stronger pre-operative grip strength and younger age were independent predictors of higher post-operative satisfaction. These results can be used in pre-operative counseling and management of post-operative expectations.

## Background

Carpal tunnel syndrome (CTS) is the most common peripheral neuropathy[ [1]. Its prevalence reported between 1 to 16% in the general adult population [2, 3].

CTS is characterized by numbness or tingling in the sensory distribution of the median nerve. In some cases, CTS can be accompanied by pain and/or weakness of the thenar muscles which could affect thumb abduction and opposition [4].

Indicators of the disease are varied and include a combination of symptoms (e.g., paresthesia, tingling, and numbness), signs (e.g. Durkan’s sign, Phalen’s sign and Tinel’s sign) and electrophysical studies [1, 5, 6]. CTR is effective in most cases.

A limited number of studies have highlighted the predictors of patients’ outcomes and their satisfaction following CTR [3–5, 7]

Identifying preoperative predictors of clinical outcomes and post-operative satisfaction provides more information for surgical planning and preoperative consultation [5]. The present study had two aims. The primary purpose of this study was to identify predictors of clinical outcomes after CTR. The secondary objective of this study was to evaluate patients’ satisfaction and correlated predictors following CTR.

## Methods

**Fig. 1:**
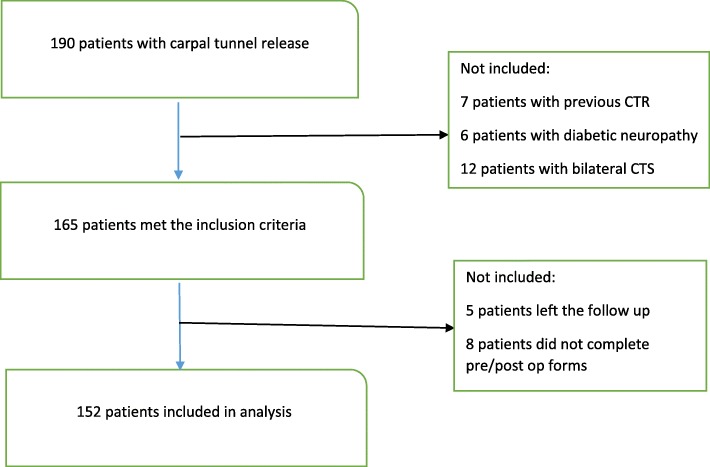
Flowchart of the study population

This prospective study was conducted in 2018. A sample of 152 patients with carpal tunnel syndrome who referred to the Imam Reza hospital, Kermanshah, Iran between April 2015 and April 2017, were included. Inclusion criteria were: age over 18 years, disease duration of at least 6 months and failure in medical treatment. Patients with a history of previous wrist surgery or trauma and those with diabetic neuropathy and cases with bilateral CTS were excluded. Moreover, 5 patients (3.03%) left the follow up and 8 subjects (4.84%) did not complete pre/postoperative forms [Fig. 1].

The present study was approved by the Scientific Research Board of the Kermanshah University of Medical Sciences. Informed written consent was obtained from all patients before enrolment.

Meticulous clinical examinations including specific provocative tests of the hand (Phalen’s sign, Tinel’s signs, and Durkan’s tests), the Semmes–Weinstein monofilament test, and grip strength test were performed.

We performed the Semmes–Weinstein monofilament test at the most prominent finger for each patient. Results were recorded by a 5-point scale as follows: 1- normal (2.83 monofilaments), 2-diminished light touch (3.61), 3- diminished protective sensation (4.31), 4- loss of protective sensation (4.56), and 5-untestable sensation (6.65) [8]. Grip strength test was conducted using a Jamar dynamometer (Sammons Preston, Bolingbrook, Illinois) [3].

Electrophysiological tests were conducted pre-operatively for each patient. Sensory nerve action potential (SNAP) (in μv), the peak latency of SNAP (in ms), the peak amplitude of compound muscle action potential (in mV), and conduction velocity of the SNAP (in m/s) were recorded. According to the American Association of Electrodiagnostic Medicine criteria [8] patients were categorized into three groups (mild, moderate and severe).

The Boston Carpal Tunnel Questionnaire (BCTQ) were assessed at intake and last follow -up visits to evaluate clinical outcomes.

Boston Carpal Tunnel Questionnaire (BCTQ) scores were recorded at preoperative visits and six-month post-operative visits. The BCTQ [6] is a disease-specific tool that can be used to assess symptom severity, functional status, and clinical outcome of patients with CTS. BCTQ is comprised of two separate parts: a symptom severity scale (SSS) and a functional status scale (FSS). The first part comprises 11 questions (Q1 ~ Q11) concerning the severity of pain, tingling, numbness, and weakness. The functional status scale has 8 questions of activities of daily tasks. Each item scoring from 1 to 5 in the ascending order making a total score of 55 for SSS (with 11 being the best and 55 being the worst) and 40 for the FSS (with eight being the best and 40 being the worst). Mean Boston score for symptom severity and mean Boston score for functional status were obtained by dividing the total SSS by11 and total FSS by 8. BCTQ was translated into the Persian language and it was validated. All patients were managed with the minimal invasive open technique for carpal tunnel release under local anesthesia.

Age, sex, hand dominancy, smoking, symptoms duration, body mass index (BMI), the Semmes–Weinstein monofilament test, grip strength, electrophysiological severity, scores of the Boston Carpal Tunnel Questionnaire, presence of positive physical examination signs (Phalen’s sign, Tinel’s signs and Durkan’s tests), presence of thenar atrophy and EMG abnormalities (fibrillations, positive sharp waves or fasciculations) were selected as probable predictors of clinical outcomes and patients’ satisfaction.

Patients’ satisfaction was determined by a 10-point verbal descriptor nominal scale (1 = very poor, 5 = fair, 10 = excellent) and recorded, at last, follow -up visits.

### Statistical analysis

Data analysis was performed with SPSS 21 (SPSS Inc. Chicago, Illinois). The mean and standard deviation of quantitative variables were calculated. The normality of quantitative variables was checked by the Kolmogorov -Smirnov test. The Wilcoxon test was used to compare preoperative SSS and FSS with 6- month postoperative SSS and FSS. Because the variables of pre-operative SSS, pre-operative FSS, Satisfaction, as well as change scores in SSS and FSS were non-normal, we used nonparametric tests such as Mann-Whitney-U test, Kruskal Wallis, and Spearman correlation test for checking the relationship between mentioned variables with other variables and those that were significant entered into the linear regression model. It is notable, because dependent variables (pre-operative SSS, pre-operative FSS, Satisfaction, change score in SSS, and change score in FSS) were non-normal we take their logarithm (Ln) before doing regression tests. Significant level < 0.05 considered as the significant level. *P* values < 0.05 considered as the significant level.

## Results

There were 118 (77.6%) females and 34 (22.36%) males. The mean age was 50.50 ± 7.24 years. Sixteen (10.5%) patients had CTS symptoms for less than 1 year, 123(80.9%) patients had symptoms for a period of 1 to 4 years, and 13(8.6%) of our patients were symptomatic for more than 4 years. In 69.7% of patients (106 individuals), the dominant hand was involved. [Table 1].

**Table 1:** personal characteristics of the subjects

Variables		Frequency	Frequency percent
Gender	Male	34	22.4
	Female	118	77.6
Duration of symptoms	One year	16	10.5
	1–4 year	123	80.9
	> four years	13	8.6
Electrophysiological severity	Mild	8	5.3
	Moderate	72	47.4
	Sever	72	47.4
Involved side	Dominant	106	69.7
	Non dominant	46	30.3
Thenar muscle atrophy	Absent	132	86.8
	Present	20	13.2
Smoking	No	129	84.9
	Yes	23	15.1
Hypothyroidism	No	143	94.1
	Yes	9	5.9
Phalen test	Positive	135	88.8
	Negative	17	11.2
Tinel test	Positive	140	92.1
	Negative	12	7.9
Durkan test	Positive	141	92.8
	Negative	11	7.2
EMG abnormality	Absent	137	90.1
	Present	15	9.9
Monofilament test	Normal	8	5.3
	Diminished light touch	50	32.9
	Diminished protective sensation	92	60.5
	Loss of protective sensation	2	1.3
	Loss of protective sensation	2	1.3
Previous carpal injection	Yes	61	40.13
	No	91	59.86

### Predictors of pre-operative BCTQ (SSS & FSS)

The results of univariate analyses showed that the pre- operative SSS was related to age, duration of symptoms, electrophysiological severity, and the presence of thenar muscle atrophy(*P* < 0.05) [Table 2]. As well as, age, duration of symptoms, and the presence of thenar muscle atrophy were related to the pre-operative FSS (*P* < 0.05) [Table 2]. According to the linear regression model, duration of symptoms and electrophysiological severity were the independent predictors of preoperative SSS. The model predicted 63% of the variance of pre-operative SSS [Table 3]. Meanwhile, linear regression model showed the age, duration of symptoms, and the presence of thenar muscle atrophy as the predictors of pre-operative FSS [Table 4].

**Table 2:** the relationship between various variables with pre-operative SSS, pre-operative FSS, satisfaction, and change scores in SSS and FSS

Variables	Pre-operative SSS	Pre-operative FSS	Change score in SSS	Change score in FSS	Satisfaction
Age	*r* = 0.210 *P* = 0.009*	*r* = 0.197 *P* = 0.015*	*r* = −0.196 *P* = 0.016*	*r* = 0.226 *P* = 0.005*	*r* = −0.193 *P* = 0.017*
Sex	Z = 0.461 *P* = 0.645	Z = -0.586 *P* = 0.558	Z = -0.458 *P* = 0.647	Z = -1.243 *P* = 0.214	Z = -0.638 *P* = 0.524
BMI	*r* = 0.087 *P* = 0.287	*r* = 0.005 *P* = 0.95	*r* = 0.037 *P* = 0.186	*r* = 0.044 *P* = 0.31	*r* = −0.006 *P* = 0.937
Duration of symptom	K2 = 8.006 *P* = 0.018*	K2 = 8.007 *P* = 0.018*	K2 = 8.093 *P* = 0.017*	K2 = 2.638 *P* = 0.267	K2 = 0.725 *P* = 0.696
Electrophysiological Severity	K2 = 99.896 *P* < 0.001*	K2 = 1.894 *P* = 0.388	K2 = 99.786 *P* < 0.001*	K2 = 2.927 *P* = 0.231	K2 = 2.69 *P* = 0.260
Involved side	Z = -0.339 *P* = 0.739	Z = -0.437 *P* = 0.662	Z = -0.359 *P* = 0.719	Z = -0.594 *P* = 0.552	Z = -0.178 *P* = 0.859
Grip strength	*r* = 0.023 *P* = 0.779	*r* = 0.03 *P* = 0.71	*r* = 0.020 *P* = 0.805	*r* = 0.063 *P* = 0.443	*r* = 0.655 *P* < 0.001*
Thenar muscle atrophy	Z = -3.039 *P* = 0.002*	Z = -2.392 *P* = 0.017*	Z = --3.084 *P* = 0.002*	Z = -1.072 *P* = 0.284	Z = -1.561 *P* = 0.119
Smoking	Z = -0.533 *P* = 0.594	Z = -0.240 *P* = 0.810	Z = -0.497 *P* = 0.619	Z = -0.067 *P* = 0.947	Z = -0.497 *P* = 0.619
Hypothyroidism	Z = -1.345 *P* = 0.176	Z = -0.730 *P* = 0.466	Z = -1.306 *P* = 0.192	Z = -0.145 *P* = 0.885	Z = -0.057 *P* = 0.955
Phalen test	Z = -1.201 *P* = 0.230	Z = -1.624 *P* = 0.104	Z = -1.066 *P* = 0.287	Z = -1.766 *P* = 0.077	Z = -0.371 *P* = 0.710
Tinel test	Z = -0.88 *P* = 0.379	Z = -0.390 *P* = 0.696	Z = -0.859 *P* = 0.390	Z = -0.531 *P* = 0.595	Z = -0.423 *P* = 0.672
Durkan test	Z = -1.439 *P* = 0.150	Z = 1.126 *P* = 0.260	Z = -1.385 *P* = 0.166	Z = -0.790 *P* = 0.430	Z = -0.130 *P* = 0.897
EMG abnormality	Z = -0.365 *P* = 0.715	Z = -0.225 *P* = 0.822	Z = -0.381 *P* = 0.704	Z = -0.627 *P* = 0.531	Z = -0.415 *P* = 0.678
Monofilament test	K2 = 0.445 *P* = 0.931	K2 = 7.56 *P* = 0.056	K2 = 0.604 *P* = 0.896	K2 = 4.705 *P* = 0.195	K2 = 4.780 *P* = 0.189
Previous carpal injection	Z = 3.71 *P* = 0.151	Z = 1.18 *P* = 0.554	Z = 3.881 *P* = 0.275	KZ = 7.50 *P* = 0.067	Z = 3.861 *P* = 0.277

**Table 3:** predicting pre-operative SSS by variables of age, duration of symptoms, electrophysiological severity, and thenar muscle atrophy

Predictors	B	Standard error	Sig.	Standard coefficient Beta	CI 95%
Constant	2.903	0.080	< 0.001	–	2.746–3.060
Age	0.002	0.001	0.111	0.08	0.001–0.004
Duration of symptoms	0.053	0.019	*0.007	0.134	0.015–0.019
Electrophysiological severity	0.225	0.015	* < 0.001	**0.776**	0.195–0.255
Thenar muscle atrophy	−0.006	0.026	0.822	−0.012	−0.059-0.046
Model summary					
Model	R	R2	Adjusted R2		
Age, duration of symtoms, electrophysiological severity, thenar muscle atrophy	0.804	0.647	0.637		

Dependent variable: Ln SSS pre-operation

**Table 4:** predicting pre-operative FSS by variables of age, duration of symptoms, and thenar muscle atrophy

Predictors	B	Standard error	Sig.	Standard coefficient Beta	CI 95%
Constant	0.937	0.121	< 0.001	–	0.697–1.176
Age	0.004	0.002	*0.020	0.185	0.001–0.008
Duration of symptoms	− 0.063	0.030	*0.037	−0.165	0.122–0.004
Thenar muscle atrophy	0.081	0.039	*0.037	0.165	0.005–0.157
Model summary					
Model	R	R2	Adjusted R2		
Age, duration of symtoms, thenar muscle atrophy	0.309	0.095	0.077		

### Predictors of change scores in SSS and FSS

Overall, the result of the present study revealed that the surgery improved outcomes [Table 5]. Most variables did not have strong predictive power in clinical outcomes [Table 2]. However, duration of symptoms and electrophysiological severity were predictors of the change score in SSS (*P* < 0.05) [Table 6]. Moreover, age was the only predictor of change scores in FSS(*P* < 0.05) [Table 7].

**Table 5:** comparison of pre and post- operative SSS and FSS variables by Wilcoxon test

Variables		Mean rank	Mean + SD	Statistical test
SSS	Pre-operative	76.33	38.65 ± 6.22	Z = -10.69 *P* < 0.001
	Post-operative	0.00	1.29 ± 0.48	
FSS	Pre-operative	73.50	3.10 ± 0.49	Z = -10.80 *P* < 0.001
	Post-operative	0.00	1.68 ± 0.48

**Table 6:** predicting change score in SSS by variables of age, duration of symptoms, and electrophysiological severity

Predictors	B	Standard error	Sig.	Standard coefficient Beta	CI 95%
Constant	2.844	0.084	< 0.001	–	2.678–3.011
Age	0.002	0.001	0.134	0.077	−0.001-0.004
Duration of symptoms	0.056	0. 20	*0.007	0.137	0.015–0.096
Electrophysiological severity	0.231	0.016	* < 0.001	0.767	0.199–0.263
Thenar muscle atrophy	−0.003	0.028	0.908	−0.006	−0.58-0.052
Model summary					
Model	R	R2	Adjusted R2		
Age, duration of symtoms, electrophysiological severity, Thenar muscle atrophy	0.796	0.634	0.624

**Table 7:** predicting change score in FSS by variable of age

Predictors	B	Standard error	Sig.	Standard coefficient Beta	CI 95%
Constant	−0.193	0.211	0.362	–	−0.610-0.224
Age	0.010	0.004	0.015	0.201	0.002–0.018
Model summary					
Model	R	R2	Adjusted R2		
VAge	0.201	0.041	0.034		

### Predictors of post-operative satisfaction

At first, we assessed the relationship between post-operative satisfaction and suggested variables. The variables of age and preoperative grip strength were correlated with the patients’ satisfaction (*P* < 0.05) [Table 3]. In the next step, we conducted a linear regression model. Interestingly, the result of the regression model showed the preoperative grip strength as the powerful predictor of post-operative satisfaction (B = 0.026, *P* < 0.001). However, the model did not show such a strong predictive power for age (B = − 0.003, *P* = 0.043). Overall, the model predicted 73% of the variance of post-operative satisfaction [Table 8].

**Table 8:** predicting satisfaction by grip strength and age by linear regression test

Predictors	B	Standard error	Sig.	Standard coefficient Beta	CI 95%
Constant	1.355	0.048	< 0.001	–	1.26–1.45
Grip strength	0.026	0.002	< 0.001	0.77	0.023–0.030
Constant	1.49	0.85	< 0.001	–	1.33–1.66
Grip strength	0.026	0.002	< 0.001	0.759	0.023–0.030
Age	−0.003	0.001	0.043	−0.005	0.001
Model summary					
Model	R	R2	Adjusted R2		
Grip strength	0.770	0.593	0.590		
Grip strength and age	0.777	0.604	0.599		

Dependent variable: Ln satisfaction

Critical complications such as tendon, muscle or nerve damages were not observed in the study.

## Discussions

Carpal tunnel syndrome is the most common peripheral neuropathy [9]. Carpal releasing surgery proved to be effective in many cases, although reported success varies. The incidence of CTS is increasing as 11% of females and 3.5% of males, with increasing life expectancy [10].

Open release of the carpal tunnel, introduced by Phalen et al. (1950) is the standard treatment for the CTS [11]. According to a recent review of a long-term follow-up after CTS surgery, clinical success reported between 75 and 90% [1]. In spite of the considerable improvement in patients’ symptoms, their satisfaction with releasing surgery is still unpredictable. In the present study, we have evaluated the predictors of clinical outcomes and satisfaction of patients with CTR.

The Boston carpal tunnel syndrome questionnaire is a disease-specific measure [6]. The sensitivity of the Boston CTS Questionnaire for detecting a change after carpal tunnel surgery has been demonstrated [6]. Gay et al. found that the BCTQ is more sensitive to changes in clinical stats of patients than the electrophysiological findings, clinical examination or other generic questionnaires such as the Short-Form 36 and Disabilities of the Arm, Shoulder, and Hand questionnaire [12].

Some factors have been suggested as outcome predictors of carpal releasing surgery including age, gender, smoking, occupation, underlying disease, duration of symptoms, and preoperative muscle weakness or atrophy [7, 8] However, in this study, most variables did not have a strong predictive value on patients’ outcomes. In our study, patients with severe electrophysiological findings had higher postoperative FSS score. This finding suggests that early diagnosis and treatment of carpal tunnel syndrome could improve clinical outcomes.

Moreover, our results did not demonstrate a relationship between clinical outcomes and the duration of symptoms. Eisenhardt et al. reported a recovery period of 16 days in patients whose symptoms lasted less than 1 year compared to 25 days of recovery in patients with duration of symptoms more than 1 year [13]. They found that the duration of paresthesia did not have a significant effect on the outcome of CTR [13].

*Grip strength* can be decreased by *CTS significantly* which can lead to losing productivity at work and daily activities [14]. This study concluded that patients with a weaker preoperative grip strength had lower satisfaction following a CTR. Brown et al. reported that patients with reduced grip strength require longer postoperative duration to recover the grip strength and the recovery is also incomplete. They have suggested that this may be due to progressive median nerve damage in long-standing CTS [11]. Levine et al. reported that patients’ satisfaction had a moderate correlation with the improvement of the functional status score and highly with changes in the symptom severity scale score and a moderate correlation with the change of the functional status score [6].

The relationship between age and patients’ satisfaction after CTR is not well documented. Results of our study revealed a correlation between age and post-operative satisfaction. Hansen and Larsen [15] reported that patients over 65 years old had less favorable results on the BCTQ after CTR. They concluded that age may have an adverse effect on nerve regeneration. Atroshi et al. found that age was a significant predictor of patient dissatisfaction [16].

### Strength and limitations

This study has shown to be powerful in three main areas: First, a large sample of patients, second, organized and detailed data on symptoms and third, a systemized physical examination.

This study had limitations. We measured patients’ satisfaction only at 6 months after CTS surgery. However, studies with longer follow-up periods showed that the persistence of positive effects had no further improvement beyond 6 months [4, 17, 18].

Furthermore, there was a substantial drop-out rate among patients between the intake and the follow-up at 6 months.

Conclusions: Results of the present study revealed that there was a significant improvement in clinical outcomes after CTS surgery. Stronger pre-operative grip strength and younger age were independent predictors of higher post-operative satisfaction. These results can be used in pre-operative counseling and management of post-operative expectations.

## Data Availability

All data are available from the corresponding author upon reasonable request.

## Abbreviations

BCTQ: Boston Carpal Tunnel Questionnaire
CTR: Carpal Tunnel Release
CTS: Carpal Tunnel Syndrome
CTS: Symptom Severity Scale
FSS: Functional Status Scale
